# Commentary on “Mandible exosomal ssc-mir-133b regulates tooth development in miniature swine via endogenous apoptosis”

**DOI:** 10.1038/s41413-018-0033-8

**Published:** 2018-10-17

**Authors:** J. Edward Puzas

**Affiliations:** University of Rochester School of Medicine and Dentistry, Rochester, NY USA

This report, “Mandible exosomal ssc-mir-133b regulates tooth development in miniature swine via endogenous apoptosis” by Li et al. is an important step forward in describing the factors that control tooth development in a large animal model. That many of the regulatory miRNA pathways have been elucidated in murine species have always begged the question as to how relevant they are in larger mammals and eventually humans. This investigation begins to bridge this issue.

Underscoring the interrelatedness of miRNA regulation, the authors first demonstrate that ssc-mir-133b is selectively expressed in different cell types in developing dental tissues. And since ssc-mir-133b expression is co-localized with an apoptotic signal that it might be closely related to the control of cell turnover during the period of early development.

Yet, the ssc-mir-133b signal does not appear to be a general regulator of apoptosis genes. The authors found through bioinformatics data that a number of regulatory intermediates are putative regulators of apoptosis, but Mcl-1 was the only member that was downregulated with ssc-mir-133b overexpression in premolar primary dental mesenchyme. Moreover, the authors present data to support that there is a conserved binding site for ssc-mir-133 on the Mcl-1 UTR.

Cellular function data appear to corroborate the ssc-mir-133b/Mcl-1 interaction in that exposure to ssc-mir-133b promoted the expression of apoptotic effectors such as caspace-3, caspace-7, and caspace-9. In this same system overexpression of Mcl-1 reversed this process. All of these results suggest that Mcl-1 may, indeed, be a key target pathway for ssc-mir-133b.

Perhaps the most interesting and meaningful observation in this system is the inter-tissue mechanisms by which apoptotic signals are transmitted. The authors found that in early tooth development there are no apoptotic signals in pre-molar tissues but that mandibular tissues showed demonstrable signs of apoptosis. As the tooth structure matured, signals for apoptosis appeared in the dental mesenchyme. This led the investigative team to propose a “transmission” signal from mandibular cells to tooth germal cells that further regulates their development. To delve deeper into the mechanism of this transmission the authors separately isolated mandible and tooth germ tissues and then prepared exosomes from the mandibular tissue. Exosome characterizations showed high levels of ssc-mir-133b. When mandible and tooth germ cells were co-cultured in a transwell system there was evidence of signal transmission through ssc-mir-133b. This observation was mitigated in the presence of an exosome inhibitor.

As a final proof of principle that miRNA signals are key regulators in in vivo systems the authors used imaging systems in animal experiments to show that molars developed normally in control animals but were severely stunted in animals where ssc-mir-133b was overexpressed. As expected Mcl-1 rescued this effect.

In summary, this report provides credible evidence that the mandible provides key apoptotic signals through the expression of a mandible-specific miRNA, ssc-mir-133b, that controls tooth development. It also adds further evidence that there is both a spatial and temporal regulation of germal cell populations through the regulation of mitochondrial mediated apoptosis and that exosomes play a key role in this paracrine-type effect by virtue of their ability to transmit key miRNAs. The final figure in this report is a diagrammatic representation of this spatio/temporal regulatory pathway. Not only do the findings in this paper discover novel regulatory mechanisms in tooth development but they also add to our understanding of how non-protein regulators can be important factors in cell-cell communication.

